# Plant Bioactive Compounds of Brazilian Pampa Biome Natural Grasslands Affecting Lamb Meat Quality

**DOI:** 10.3390/foods13182931

**Published:** 2024-09-16

**Authors:** Luiza Jacondino, Cesar Poli, Jalise Tontini, Gladis Correa, Itubiara da Silva, André Nigeliskii, Renius Mello, Angélica Pereira, Danielle Magalhães, Marco Trindade, Sandra Carvalho, James Muir

**Affiliations:** 1 Department of Animal Science, Federal University of Rio Grande do Sul, Av. Bento Gonçalves 7712, Porto Alegre 91540000, RS, Brazil; lurodegheri@hotmail.com (L.J.); jsetontini@yahoo.com.br (J.T.); tubmaciel@hotmail.com (I.d.S.); 2 Department of Animal Science, Federal University of Pampa, R. Vinte e Um de Abril, 80, Dom Pedrito 96450000, RS, Brazil; gladiscorrea@unipampa.edu.br; 3 Department of Science and Food Technology, Federal University of Santa Maria, Av. Roraima, 1000, Santa Maria 97105900, RS, Brazil; fogaca.zootecnia@hotmail.com (A.N.); reniusmello@gmail.com (R.M.); 4 Department of Food Engineering, University of Sao Paulo, Av. Duque de Caxias Norte, 225, Pirassununga 13635970, SP, Brazil; angelpereira@usp.br (A.P.); d.magalhaes@yahoo.com.br (D.M.); trindadema@usp.br (M.T.); 5 Department of Animal Science, Federal University of Santa Catarina, Rodovia Admar Gonzaga, 1346, Florianopolis 88034000, SC, Brazil; sandra.carvalho@ufsc.br; 6 Texas AgriLife Research, Texas A&M University, 1229 N. US Hwy 281, Stephenville, TX 76401, USA; jim.muir@ag.tamu.edu

**Keywords:** antioxidants, *Desmodium incanum*, sensory analysis, native legumes, secondary compounds, tannin, tocopherol

## Abstract

Our study investigated how different levels of antioxidants and contrasting proportions of native legumes in the diet affect lamb meat quality. Twenty-four male Texel lambs were randomly assigned to three groups: two groups on a natural pasture in southern Brazil (Pampa Biome), each at a different proportion of legumes: Low-legume (LL, 4.37%) and High-legume (HL, 14.01%); the other group was stall-fed (Control) to achieve the same growth rates as the grazing groups. Cold carcass yield from the Control lambs was higher than HL. The meat from pasture-fed animals had a higher deposition of muscle α-tocopherol and lower lipid oxidation (TBARS values) after 9 days of storage. LL lambs had higher subcutaneous fat thickness, which promoted better sensory quality of the meat, as assessed by a trained panel. Pasture-based diets enhanced odd- and branched-chain fatty acids (OBCFAs), trans vaccenic acid, and total conjugated linoleic acids (CLAs), while decreasing elaidic acid. Despite the lower ∆9-desaturase activity, the higher proportion of *Desmodium incanum* (condensed tannin-rich native legume) in the HL diet did not impact meat nutritional quality. Finishing lambs on the Pampa Biome grasslands is an option for improving the oxidative stability and beneficial fatty acid content of lamb meat, which improves product quality and human consumer health.

## 1. Introduction

Consumers are increasingly interested in obtaining quality lamb meat from sustainable agricultural practices [1]. Finishing diet and country of origin are very important for meat consumers’ purchasing decisions, with a preference for locally produced meat and grass-fed animals [2]. Well-managed grazing systems can improve soil health, help sequester carbon, maintain plant biodiversity, and reduce animal welfare issues [3]. The demand for “healthy” products produced with environmental sustainability and animal welfare has increased interest in extensive lamb production systems [4].

Brazil is made up of six biomes, each with distinct characteristics. Among them, the Pampa Biome covers more than half of Rio Grande do Sul State (~69%) and extends into Argentina and Uruguay. It is the second smallest Brazilian biome, occupying only 2.3% of the country’s total surface, but it stands out for housing 9% of the country’s biodiversity [5], with an extremely high vegetation diversity. In a single plot of 1 × 1 m in Brazilian Pampa Biome grasslands, Menezes et al. [6] found 56 species of vascular plants. As it is a natural pastoral ecosystem, livestock farming is the best option for sustainable use for food production purposes. For their preservation, adequate management must be carried out, and the products from these pastures must be valued [7]. 

In southern Brazil, lamb meat production is carried out mainly in pastoral systems. The Rio Grande do Sul has the third largest sheep herd in the country; the main producing municipalities are in the south and southwest of the state [8], that is, in the Pampa Biome area. This vegetation, containing representatives of several botanical families, produces forage rich in secondary compounds, and this diverse diet gives unique characteristics to the animal product obtained there [7]. Among the bioactive compounds found in forages, α-tocopherol (vitamin E) [9,10] and condensed tannins (CTs) [11,12] are highlighted for their potential to improve meat quality due to their antioxidant characteristics. 

Recently, Tontini et al. [13] found large secondary compound amounts in natural pastures in southern Brazil, with emphasis on *Desmodium incanum*, a native legume with high leaf CT levels. Furthermore, the inclusion of legumes in pastures optimizes forage protein values and promotes better overall forage ruminal digestibility [14]. Although there are many reports about the diversity and quality of natural pastures in the Pampa Biome, few studies have compared its lamb meat quality with more common systems such as confinement, with low levels of diet antioxidants. There is a lack of research on the influence of bioactive compounds and native legumes in this ecosystem as it relates to lamb meat quality. 

We hypothesized that finishing lambs on a combination of legumes and grass pasture would produce carcasses and meat with greater human-consumer health traits when compared to those finished in a feedlot system. Therefore, the objective of this study was to characterize lamb meat quality produced in natural pastures of the Pampa Biome (with a low or high proportion of legumes), including sensory acceptability to humans, compared to traditional stall-fed lamb meat as a Control.

## 2. Materials and Methods

The experiment was conducted in two locations. Finishing lambs on natural pastures was carried out at the Agronomic Experimental Station of Universidade Federal do Rio Grande do Sul (UFRGS), Pampa Biome region of the state of Rio Grande do Sul—RS, Brazil, from 19 January to 30 March 2022. Lamb finishing in confinement was developed at the Sheep Experimental Unit of Universidade Federal do Pampa (UNIPAMPA), Dom Pedrito Campus, Brazil, from 27 December 2021 to 30 March 2022. This study was approved by the university’s Animal Experimentation Ethics Committee of UFRGS (protocol number 36468), the Animal Experimentation Ethics Committee of UNIPAMPA (protocol number 23100.001885/2022-55), and the Human Research Ethics Committee of the Faculty of Animal Science and Food Engineering at the Universidade de São Paulo under the number CAAE: 58412822.0.0000.5422. 

### 2.1. Animals and Production Systems

Twenty-four castrated male Texel lambs of the same ranch (same pasture type and genetic origin), born between July and August 2021, were raised together in a natural pasture in southern Brazil until weaning, which occurred at approximately 150 days of age and an initial live weight of 28.36 ± 1.37 kg. After weaning, the animals were distributed to achieve a similar average weight per treatment, into three treatments in a completely randomized experimental design. These included stall-fed lambs (Control, n = 7); lambs finished on natural pastures of the Pampa Biome with a low proportion of legumes (LL = Low-legume group, n = 8); and lambs finished on natural pastures of the Pampa Biome with a high proportion of legumes (HL, High-legume group, n = 9). Before the start of the experiment, all animals received an anthelmintic treatment based on levamisole phosphate (Ripercol^®^ 10%, 5 mg/kg, Zoetis, Porto Alegre, RS, Brazil). Water and salt blocks (Blokus^®^ ovinos, Supra, Porto Alegre, RS, Brazil) were always available for all of the animals.

#### 2.1.1. Stall Feeding System

A group of lambs were housed in individual pens (3 m^2^) and were not allowed to access the pasture. In the first 10 days, the lambs went through a period of adaptation to the pens and the diet. After this period, they received a diet composed of grass hay and concentrate (corn, soybean meal, and calcareous). The stall-fed treatment was the Control group, with a diet low in antioxidants. The food supplied to the stall group was adjusted when the growth rate did not correspond to that of the lambs in the pasture groups, measured by weighing every 14 days. The amount of concentrate in the diet varied according to average daily gain. During the experiment period, three different diets were provided: from 0 to 54 d the animals received a diet with roughage: concentrate ratio of 80:20; from 55 to 76 d 75:25; and from 77 to 93 d (slaughter) 60:40. Feed quantities offered and refused were recorded daily to measure dry matter intake (DMI).

#### 2.1.2. Grazing Systems 

The other two groups of lambs were allowed to continuously graze on natural pastures typical of the Pampa Biome (Central Depression region of RS) with a minimum forage supply of 12% of live weight. In the Low-legume (LL) treatment, three herbicide applications (2.4D; 2.4D and metsulfuron-methyl and 2.4 D and picloram) were carried out to suppress broadleaf species. Each treatment had an area of 1.3 ha and access to shade. The animals remained in this area for 70 days until slaughter. Mechanical mowing was carried out 48 days after the beginning of the experiment for weed control and to maintain a similar sward structure between both treatments. 

Plant species coverage (botanical composition) was estimated in three periods (before grazing, grazing period, and end of grazing). Species composition data were obtained in 9 plots (1 m^2^) per treatment, randomly distributed in the paddocks. In each plot, all vascular plant species were identified, and their coverage was estimated using the Londo decimal scale [15]. Whenever necessary, plant samples were collected for subsequent taxonomic identification. Species classification into families was carried out according to Stevens [16], and the nomenclature of species was as described by Boyle et al. [17]. In addition to estimating species coverage, the structural attributes of the vegetation were also measured: average vegetation height, estimated cover of mantle, dung, and bare soil. 

In the Pampa Biome natural grassland system, the species that contributed the most forage mass (FM) in the LL treatment were: *Eragrostis plana* (54.23%), *Mnesithea selloana* (8.31%), *Paspalum dilatatum* (5.65%), *Eryngium horridum* (4.99%), *D. incanum* (4.37%), *Cynodon dactylon* (2.86%), *Cyperus aggregatus* (2.77%), *Carex bonariensis* (1.88%), *Paspalum notatum* (1.37%), and *Pfaffia tuberosa* (0.8%). In the HL treatment, species included *Eragrostis plana* (23.79%), *D. incanum* (14.01%), *Paspalum dilatatum* (6.45%), *Carex phalaroides* (4.91%), *Cynodon dactylon* (4.59%), *Eryngium horridum* (4.3%), *Carex bonariensis* (3.37%), *Cyperus aggregatus* (3.04%), *Paspalum notatum* (3.0%), *Pfaffia tuberosa* (1.34%), and *Sida rhombifolia* (0.81%). 

Forage mass and forage accumulation rate assessments were carried out in January, February, and March. Forage mass (FM) was performed by 15 standard evaluations in randomly selected 0.25 m^2^ quadrats. A subsample was taken for separation into leaf, stem, inflorescence, *D. incanum*, other broad leaves, or dead material and dried in a forced-air oven at 65 °C until constant weight. Then, the material was weighed on a digital scale to evaluate the dry matter yield (DMY; kg/ha) of each component. Pasture height was calculated weekly as an average of 150 random measurements per paddock. A 1.5 m sward stick was used to measure the highest point of the leaf in relation to the ground [18]. In addition, six grazing-exclusion cages per paddock were used to estimate herbage accumulation rate (kg DM/ha/d). These values were determined by the difference between the initial and final DM present in the exclusion cages, divided by days of period. Pasture characteristics are presented in Table 1.

Samples from pasture were collected every 28 d using the grazing simulation technique [19]. At that time, leaves of the most common legume species in the area, *D. incanum*, were also collected. A part of the sample was immediately frozen after collection in a nitrogen cylinder (−196 °C) to prevent oxidation and was used to analyze condensed tannins and tocopherols. The other part of the sample was dried in an oven at 45 °C until constant weight for subsequent bromatological, digestibility, and carbon isotope analyses.

Grazing lamb DMI was estimated from the collection of total fecal production, using collection bags on five animals per treatment (medium-weight lambs), for five consecutive days. This evaluation was carried out 28 d after the start of the experiment and 30 d after the first evaluation. Fresh feces were removed from the bags in the morning and afternoon and weighed on a digital scale (Toledo, prix 3 Light, Porto Alegre, RS, Brazil). A sample of approximately 20% of the total (160 g) was taken, placed in aluminum trays, and dried in an oven at 70 °C to determine DM. These samples were also used for carbon isotope analysis.

All lambs (stall-fed and pasture-fed) were weighed on a digital scale every 14 d, with a 12 h fast excluding solids and liquids. Lamb body condition score was evaluated monthly, varying on a scale from 1 (very thin) to 5 (very fat). The color of the ocular mucosa was determined using the Famacha method, and the number of fecal egg counts was recorded to monitor parasitic infection. For this, a fecal sample was taken directly from the rectal ampoule of each animal and analyzed according to the methodology described by Gordon and Whitlock [20]. The samples were preserved in refrigeration (4 °C) until the tests were carried out.

### 2.2. Diet Analysis 

Hand-plucked forage samples in the pasture-based treatments and by total collection in the stall-fed treatment were analyzed to determine the chemical composition, tocopherols, CTs, and carbon isotopes (Table 2). Dry matter (DM), mineral matter (MM or ash), ether extract (EE), and crude protein (CP) were analyzed according to AOAC [21], and neutral detergent fiber (NDF) and acid detergent fiber (ADF) according to Van Soest et al. [22]. Digestible organic matter (DOM) was obtained by incubation in bovine rumen for 48 h [23]. For forage, this value can be considered equivalent to the total digestible nutrient content. 

For vitamin E analysis, alpha (α) and gamma (γ) tocopherols were extracted and analyzed in diets according to the methodology of Prates et al. [24]. The tocopherol assay was carried out in the same way as described for meat. Condensed tannin fractions were assayed in freeze-dried and ground samples of the diet as described by Tontini et al. [13]. 

Stable isotope analysis was performed as described by Pereira Neto et al. [25]. Samples of diet and feces were ball-milled using a Mixer Mill MM400 (Retsch, Newton, PA, USA) at 25 Hz for 9 min, to reduce the particle size under 100 μm before the stable isotope analyses. δ^13^C denotes a difference measurement made relative to a standard during actual analysis. δ^13^C was estimated as follows: δ^13^C = [(R Sample/R Standard) − 1] × 1000, and the results were expressed in ‰. Plant C sources from fecal samples were identified by the two-mixing pool [26]: f1 = (δ^13^C Sample − δ^13^C Source 2)/(δ^13^C Source 1 − δ^13^C Source 2); f1 + f2 = 1 or f1 = 1 − f2 where f1 represents the fraction (contribution) of source 1 (C4 species) and f2 of source 2 (C3 species).

### 2.3. Slaughtering Procedures 

All lambs were slaughtered on the same day when they reached an average final weight of 34.46 ± 1.33 kg, approximately 80 days after the start of the experiments, in a commercial abattoir (Frigorífico Specht, Salvador do Sul, RS, Brazil). This followed the standards of the Regulation of Industrial and Sanitary Inspection of Products of Animal Origin—RIISPOA [27]. After 12 h of fasting, the animals were stunned with a penetrating pneumatic pistol and bled by sectioning the great vessels of the neck. After the carcasses were kept at 4 °C for 24 h, cold carcass weight was recorded. The muscular conformation [28] and fatness of the carcasses [29] were visually evaluated.

The *Longissimus thoracis et lumborum* (LTL) muscle was removed from both sides of the carcass. The left side was completely removed, vacuum packed, and frozen at −20 °C for subsequent sensory analysis. The right side was sectioned into five portions approximately 2 cm thick, vacuum packed (Selovac, Monovac BL II, São Paulo, Brazil), and kept at −20 °C for physical, chemical, fatty acid profile, and lipid oxidation (TBARS) analyses. The last portion was wrapped in aluminum foil and preserved in an ultra-freezer (−80 °C) until analysis of tocopherols and cholesterol levels was carried out. 

### 2.4. Meat Quality Measurements 

The pH was measured directly on the carcass, between the 12th and 13th ribs, using a portable pH meter (Hanna HI 99,163 model, Hanna Instruments, Eibar, Spain). Ribeye area and subcutaneous fat thickness (SFT) were also measured at the 12th to 13th rib interface following the method described by the American Meat Science Association [30]. The color was measured with a Minolta surface spectrocolorimeter (model CM-508d, SCI setting, illuminant D65, and viewing angle 2, São Paulo, Brazil). The meat was exposed to air for at least 30 min at 15 °C before color measurement. Color values of lightness (L*), redness (a*), and yellowness (b*) were collected in three replicates per point, at three different points on the surface of the LTL muscle. 

Water-holding capacity (WHC) was measured using the methodology described by Hamm [31]. For the procedure, 0.5 g cubes of meat were placed between two circular filter papers and pressed between glass plates, weighing 10 kg for 5 min. After compression, the sample was weighed and, by difference, the amount of water lost was calculated. The result was expressed as a percentage of water exuded in relation to the initial weight of the sample.

To determine cooking loss, meat samples were packaged in laminated paper, cooked on a metal plate heated on both sides, preheated, and set to 180 °C, remaining for 4 min on each side of the sample, for a total of 8 min of cooking or until reaching an internal temperature of 82 to 85 °C. After cooking, the samples were removed from the laminated paper and cooled on absorbent paper at room temperature. Sample weight losses were determined before and after cooking. The difference between the initial weight (uncooked) and the final weight (cooked weight) corresponded to weight loss due to cooking [32]. Subsequently, the cooked samples were used for shear force analysis.

To evaluate shear force (WBSF), the TAXT plus texturometer equipped with a Warner Bratzler device (24 mm high, 8 mm wide) was used. The equipment was calibrated with a standard weight of 5 kg and a traceable standard. The device’s descent speed was 200 mm/min [33]. For each sample, five cores were taken in the form of parallelepipeds measuring 1 × 1 × 2 cm (height, width, and length, respectively), which were placed with the fibers oriented in the direction perpendicular to the Warner–Bratzler probe blade, and the results were expressed in kgf/cm^2^ and converted to Newtons (N).

To evaluate the meat’s chemical composition, proteins were determined using the modified Kjeldahl method [21]. The samples (1 g) were weighed on tissue paper and transferred to a Kjeldahl flask (paper + sample). An amount of 0.033 M of boric acid and 6 g of the catalytic mixture were added and heated on an electric plate in the hood until the solution turned blue–green and was free of undigested material (black dots). The sample was heated for another hour and allowed to cool. Ten drops of phenolphthalein indicator and 1 g of zinc powder were then added (to help break down large protein molecules). The flask was immediately connected to the distillation set. The conical end was dipped in 25 mL of 0.05 M sulfuric acid, contained in a 500 mL Erlenmeyer flask with 3 drops of methyl red indicator. A 30% sodium hydroxide solution was added to the flask containing the digested sample, through a funnel with a tap, until a slight excess of base was guaranteed. After boiling and obtaining 250–300 mL of distillate, the ammonium hydroxide solution was titrated directly with the 0.05 M sulfuric acid solution using methyl red.

### 2.5. Cholesterol and Tocopherols 

The analysis of cholesterol and α and γ tocopherols was performed simultaneously in the LTL muscle according to the procedure described by Prates et al. [24]. Meat samples (1 g) were saponified (11% KOH solution in 55% ethanol solution) at 80 °C for 15 min and homogenized with hexane solution with BHT (0.05 mg/mL). Tocopherols and cholesterol were extracted with n-hexane and dried under nitrogen flow. Analysis was performed using a CBM-20A Prominence High-Performance Liquid Chromatograph (HPLC) (Shimadzu, Kyoto, Japan) as described by Jacondino et al. [34]. Cholesterol was expressed as mg/100 g and tocopherols as mg/kg.

### 2.6. Lipid Oxidation (TBARSs)

To evaluate meat lipid oxidation, LTL muscle samples were removed from aluminum foil, thawed, placed in trays wrapped with PVC film, and stored at 4 °C, simulating retail conditions. Lipid oxidation was evaluated through the quantification of thiobarbituric acid reactive substances (TBARSs) and measured on days 0, 3, 6, and 9 of refrigerated storage, using a portion of LTL for each day of storage. Meat samples (5 g) were homogenized for 60 s with 15 mL of trichloroacetic acid following the methodology proposed by Sorensen and Jorgensen [35]. Absorbance readings were taken at 530 and 632 nm. A 5-point standard curve was prepared using a tetraethoxypropane solution of known concentration. The malondialdehyde (MDA) concentration of the samples was obtained with the equation provided by the standard curve. Analyses were performed in duplicate, and results were expressed as mg MDA/kg of meat.

### 2.7. Total Lipids and Fatty Acid Composition 

Samples of LTL of each treatment were previously freeze-dried and kept at −80 °C until the analysis. Lipids were extracted from muscles as described by Folch et al. [36]. Aliquots of muscle lipids were methylated separately using base (0.5 N sodium methoxide) and acid (5% methanolic HCl) reagents [37]. The FAs were quantified by gas chromatography (GC-2010 Plus, Shimadzu AOC 20i autoinjector, Darmstadt, Germany) with an SP-2560 capillary column (100 m × 0.25 mm diameter, 0.02 mm thick, Supelco, Bellefonte, PA, USA). The initial temperature was 70 °C with an increase (13 °C/min) to 175 °C, which was held for 27 min before a further increase to 215 °C (4 °C/min); the final temperature was maintained for 31 min. Hydrogen (H_2_) was used as the carrier gas at 40 cm^3^/s. The temperature of the flame ionization detector (FID) was 250 °C, the H_2_ flow rate was 40 mL/min, the airflow rate was 400 mL/min (synthetic air), the make-up gas flow rate was 30 mL/min kPa (N_2_), and the sampling rate was 40 msec. 

The FAs were identified by comparing the retention times of methyl esters in the samples with those of the FA C4-C24 (F.A.M.E. mix, Sigma^®^, Darmstadt, Germany), GLC 463 Reference Mixture Nu Check, vaccenic acid (V038-1G, Sigma^®^), linoleic acid (UC-61M 100 mg), conjugated linoleic acids (CLAs) (UC-60M 100 mg, Sigma), and tricosanoic acid (Sigma^®^) standards. The FAs were quantified by normalizing the area under the curve of methyl esters using GS Software Solutions (Version 2.42). The FA contents were expressed as a percentage of the total FA methyl ester quantified.

To evaluate the nutritional quality of the lipid fraction of the meat, the atherogenicity index (AI) and thrombogenic index (TI) were calculated according to the equation described by Ulbricht and Southgate [38]: AI = [(12:0 + (4 × 14:0) + 16:0)]/(MUFAs + n-6 PUFA + n-3 PUFA); TI = (14:0 + 16:0 + 18:0)/[(0.5× MUFA) + (0.5× n-6 PUFA) + (3× n-3 PUFA) + (n-3 PUFA/n-6 PUFA)]. Desirable fatty acids (DFAs) were measured according to Rhee [39], wherein DFAs = (MUFAs + PUFAs + C18:0). Δ9-desaturase activity was estimated using palmitic acid stearic acid (C18:0) according to Smet et al. [40] as follows: Δ9-desaturase C18 = [(C18:1 cis-9)/(C18:0 + C18:1 cis-9)] × 100.

### 2.8. Sensory Analysis 

The sensory characteristics of each meat sample were determined using quantitative descriptive analysis [41], with a trained panel. The tasters (25) were recruited among undergraduate and postgraduate students at the University of São Paulo, Brazil, who were regular consumers of lamb meat. Odor and basic taste recognition tests were carried out, as well as ordering tests using unstructured scales to familiarize candidates with the sensory analysis technique. Then, to tasters with greater sensory acuity, the network method was applied to develop descriptive terminology for lamb meat samples (Table 3) [42]. These were trained in two sessions followed by meetings to calibrate the tasters, that is, creating a perceptual memory of the parameters and improving the use of the scale until the panelists reached an agreement. Next, as described by MacKintosh et al. [43], the terminology and references used for sensory evaluation (aroma, off-aroma, tenderness, juiciness, flavor, fatty flavor, and off-flavor) were established.

After overnight thawing at 4 °C and boning, 2 cm steaks from the LTL muscle were prepared in an electric oven until the internal temperature reached 74 °C. After cooking, 2 cm^3^ cubes were cut (avoiding connective tissue and subcutaneous fat), and served to each taster, wrapped in aluminum foil, and kept warm for less than 10 min in an oven at 55 °C before tasting. The samples were served to 12 trained panelists, using the same tasters throughout the experiment.

Sensory evaluations were carried out in individual cabins illuminated by red lights. To evaluate the sensory attributes, a 9 cm scale was used, where the extreme left meant the least intense descriptor and the extreme right the most intense descriptor [44]. Raters tasted the samples in an order based on the designs described by MacFie et al. [45] to balance transition effects between samples. Eight sessions of 30 min each were carried out, tasting the meat samples using the monadic method, duly standardized and outlined.

### 2.9. Statistical Analysis

Data were analyzed considering a completely randomized experimental design. Each animal was considered as the experimental unit since all were fed ad libitum representing 7 to 9 replications. The effects of the dietary treatment on carcass traits, intramuscular FAs, cholesterol, and tocopherols were analyzed by one-way analysis of variance. Meat lipid oxidation data were analyzed as a repeated measurement over time, using the MIXED procedure of SAS^®^ 9.4 software. The fixed factors in the model were diet (stall-fed, Low-legume, and High-legume treatments), storage time (time: days 0, 3, 6, and 9), and their interaction (diet × time). The animals were included as a random effect in the model. Initial weight was used as a covariate in the analyses for carcass characteristics variables. Analysis of variance was performed, and the means were compared using the Tukey test at a significance level of 5%. For meat sensory characteristics, samples were blocked by diet and evaluator, and sessions were treated as a fixed effect without interactions with the other fixed effects. The feeding system treatment was considered a fixed effect, and the evaluator was included as a random effect in the model.

## 3. Results and Discussion

### 3.1. Grazing Study: Legume Proportion in Lamb Feces 

Both treatments in natural pastures of the Pampa Biome, with a low (LL) or high (HL) proportion of legumes, presented similar pasture height, forage mass, and forage accumulation rate during the 3 months of evaluation (Table 1). *Desmodium incanum* ground cover was the primary legume found in the experimental areas. The main grass found in both treatments was the invasive *Eragrostis plana* (“Capim annoni”).

Stable isotopes can be an important research tool to track grass/legume proportion in grazing experiments and the proportion of C_3_ (legumes) and C_4_ (grasses) species in the diet can be accurately predicted based on fecal samples using δ^13^C [25]. The carbon isotope (δ^13^C) in plants with C4 metabolism ranges between −9 and −17‰, and in legumes from −20 to −34‰ [46]. In our study, carbon isotope values found in lamb feces (Table 4) indicated that grasses were predominant in the LL diets, while HL pastures presented a higher proportion of legumes in the diet.

### 3.2. Carcass Characteristics 

The results of carcass characteristics are presented in Table 5. The initial weight, final weight, cold carcass weight, muscular conformation, and carcass fatness did not differ among treatments.

It is challenging to differentiate the direct effects of diet on meat and carcass characteristics from the indirect effects of growth rate due to variations in weight and age at slaughter [47]. To exclude this bias, lambs were finished on pasture (with a greater or lesser proportion of *D. incanum*) or fed a diet based on concentrates and grass hay, with similar growth rates throughout the period, and slaughtered at the same age and weight. Although the animals were slaughtered with similar final live weights, the Control lambs had greater cold carcass yield when compared to the High-legume group (*p* < 0.05). To achieve the same daily gain, animals raised on pasture likely need a greater intake of dry matter and, therefore, have a more developed digestive tract [47]. The subcutaneous fat thickness (SFT) was greater in the LL treatment.

### 3.3. Physical and Chemical Composition of Meat

Table 6 shows the physical and chemical variables of lamb meat for the different diets. The color descriptors, pH, shear force, ribeye area, levels of proteins, cholesterol, and muscle γ-tocopherol did not differ among treatments. Intramuscular fat (IMF) of LTL muscle did not differ among treatments, with an average of 2.49 g/100 g of muscle.

Cooking loss is one of the parameters to evaluate meat WHC and is closely related to meat juiciness [48]. Meat from stall-fed animals had a lower WHC compared with the pasture treatments (LL and HL) and a greater cooking loss when compared to the LL treatment. High cooking loss values are not desirable, as they signal that the meat is losing a lot of water during cooking, resulting in tougher meat with a lower degree of juiciness. During heating, the meat proteins denature and the cellular structures are disrupted, which strongly influences the WHC of meat [49]. Tissues with low WHC, as in the Control treatment (*p* < 0.05), lose more moisture and consequently greater weight during storage. This loss generally occurs on the muscle surfaces of the carcass exposed during storage [50]. 

Production systems affect the balance between natural antioxidants and meat oxidation [51]. Meat from pasture-fed lambs (LL and HL) contained higher amounts of α-tocopherol than the stall-fed group (Control) (*p* > 0.0001). Fresh green forage may be a good dietary source of a-tocopherol when pasture quality allows for high levels of a-tocopherol consumption [52]. In the same way, Yang et al. [53] showed that cattle grazing high-quality pasture achieved a high content of α-tocopherol in their muscles (4.4 to 5.8 ug/g muscle), like that obtained when grain-fed cattle were supplemented with supra-nutritional doses of vitamin E (2500 IU/head/day). 

Other studies have demonstrated that extensive forage-based systems provide sufficient muscle tocopherol content to maintain lamb meat quality throughout the exposure period, without the need to provide extra synthetic vitamin E [54], reaching maximum muscle tocopherol levels of 2.5 mg/kg [10], 4.35 mg/kg [55], and 5.88 mg/kg [56]. We found average levels of 5.53 mg/kg of tocopherol in the meat of lambs finished on native pastures in the Brazilian Pampa Biome. Röhrle et al. [57] reported that the content of α-tocopherol in Brazilian beef was higher (8.13 mg/kg) when compared to breeds from other countries (2.51 mg/kg on average).

### 3.4. Lipid Oxidation

**Figure 1:**
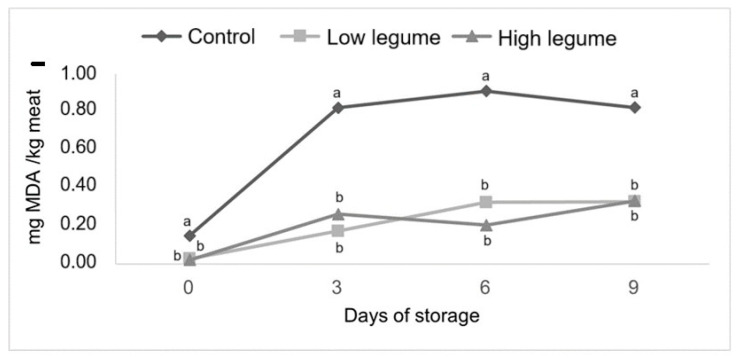
Lipid oxidation (TBARS values, mg of malondialdehyde (MDA)/kg meat) of meat from lambs finished in a pasture with a low or high proportion of legumes and in a stall-fed system with low bioactive compounds (Control) during 9 d storage. Different letters above indicate differences (*p <* 0.05) among the treatments.

Figure 1 shows lipid oxidation measured in meat from the three treatments during 9 days of refrigerated aerobic storage. Storage time affected TBARS values (*p* < 0.0001), with increasing values from day 0 to 9. Both treatments on natural pastures (LL and HL) showed lower lipid oxidation of meat when compared to the Control treatment (*p* = 0.0008). The meat from both pasture treatments was three times less oxidized than the meat from the Control.

Diet plays an important role in lipid oxidation, where animals fed concentrate have higher thiobarbituric reactive substance (TBARS) levels than animals fed pasture diet [1,51,58]. As expected, natural antioxidants (such as vitamin E) protected lamb meat against lipoperoxidation. A lower vitamin E level in the Control lambs compared with grazing lambs (1.4 vs. 5.53 mg/kg, respectively) could explain why malondialdehyde content, a marker of lipid oxidation intensity, was 0.67 mg/kg in the Control after 9 d under aerobic conditions, while it remained at 0.21 mg/kg in grazing lambs (*p* = 0.0008).

### 3.5. Fatty Acid Profile

The effects of the different lamb diets on FA composition are shown in Table 7. Total SFA contents, 12:0 (lauric acid), 14:0 (myristic acid), and 16:0 (palmitic acid) were not different among treatments. The Control group had higher 4:0 and 10:0 SFAs and lower 8:0 and 18:0 when compared to the HL group. The 10:0 increase is important because, like the cholesterol-raising FAs (SFAs from C12:0 to C16:0), it could harm human health by increasing the risk of cardiovascular disease and type-2 diabetes [59]. Whereas stearic acid (C18:0) has a neutral effect on total serum cholesterol concentration [60].

Lambs finished on pastures (LL and HL) have higher concentrations of odd- and branched-chain fatty acids (OBCFAs). These FAs have raised interest due to their positive effects on the health of the human intestinal microbiota and on several chronic diseases [61], and because they may also serve as biomarkers of rumen function [62]. Some shorter-chain BCFAs also have been reported to influence the flavor of lamb meat [63]. 

The OBCFA contents in IMF can discriminate meat samples according to the composition of the diet consumed by the lambs during the fattening period [62]. Due to the different fatty-acid composition of bacteria that digest grass (cellulolytic) or grain (amylolytic) in the rumen, a higher meat content of odd iso- and anteiso-BCFAs are associated with forage-fed lambs, whereas a higher content of unbranched odd fatty acids (OFAs) is associated with grain-fed lambs [61]. Gómez-Cortés et al. [62] suggested that forage-fed lambs would have a lower odd/anteiso FA ratio and a higher iso/(odd + anteiso) FA ratio in IMF than grain-fed ones. In agreement with this, the pasture-fed lambs in our study had a lower odd/anteiso FA ratio (LL = 5.93 and HL = 5.64, compared to the Control = 8.64) and a higher iso/(odd + anteiso) ratio (LL = 0.27 and HL = 0.29, compared to the Control = 0.20).

Total MUFAs were lower in the HL treatment than in the Control, mainly due to the lower 9c-18:1 (oleic acid). Daley et al. [60] also found that grain-fed beef produced higher concentrations of MUFAs compared to grass-fed beef, which includes FAs such as oleic acid. In the current study, lambs finished in the stall-fed system (Control) also had higher 9c-17:1 and lower 11c-20:1 compared to the other treatments, and higher 9c-16:1 compared to HL. Oleic acid was the dominant MUFA measured in the current study (71.4% of the total MUFAs), followed by vaccenic acid (4.8% of total MUFAs). 

The pasture lambs (LL and HL) had higher contents of 9t-16:1, 10t-18:1, and 11t-18:1 (vaccenic acid) and lower contents of 9t-18:1 (elaidic acid). Elaidic acid is detrimental to cardiovascular health and is typically associated with highly processed foods [64]. In contrast, vaccenic acid is considered beneficial for human health [65] as it is a precursor to the conjugated linoleic acid (CLA) 9c,11t-18:2 (rumenic acid), a potent anti-carcinogen that has several beneficial health effects [61]. In this study, rumenic acid was higher in LL, intermediate in HL, and lower in the Control (*p* < 0.0001), while total CLAs was higher in both pasture treatments (LL and HL).

A lower activity of ∆9-desaturase (C18) was found in the HL group than in the Control lambs. *Desmodium incanum* was the main legume found in the HL treatment and showed 5.31% of total condensed tannins (CTs) and 34.59 mg/g of binding CTs (Table 2). Other studies also found that the inclusion of CTs in the diet decreased the enzymatic Δ9-desaturase activity [34,66] since high PUFA and CLA contents can inhibit the expression of lipogenic enzymes, resulting in less activity. This can also be related to the lower oleic acid and 16:1 concentration found in our study since this enzyme is essential for MUFA synthesis. 

The sum of all non-conjugated dienes was higher in the LL group than in the stall-fed system (due to the greater content of t9,12c-18:2 in this treatment). There were no differences in total PUFAs, n-3 PUFA, and n-6 PUFA; only 6t-18:2 was higher in the HL treatment than in the stall-fed system.

Ratios (PUFAs/SFAs; n-6/n-3) and health indexes (AI, TI, DFAs) did not differ among treatments. The World Health Organization [67] recommends that the n-6/n-3 proportion should not exceed a value of 4 in the human diet to reduce the risk of cardiovascular diseases. In our study, this ratio was less than 2.1 in all lambs, which is well below the recommended value. Health recommendations suggest that PUFA:SFA ratios should be at least 0.4, which is a challenge to achieve in ruminants, even with forage-based diets [68].

### 3.6. Sensory Analysis

The results of the sensory analysis are presented in Table 8. Meat from pasture-fed lambs with a lower proportion of legumes (LL) showed greater tenderness, juiciness, and most intense characteristic flavor compared to the other treatments.

Meat tenderness and juiciness are positively correlated with fat content due to a direct effect of fat being softer than lean and/or an indirect effect of reducing the shortening of muscle fibers [47]. Although we did not find differences in carcass fatness or intramuscular fat among treatments, the LL lambs presented higher SFT (3.33 mm) compared to the Control and HL treatments (2.37 and 1.75 mm, respectively). The tenderness of the meat, measured by sensory analysis, may be related to the greater STF that protects the carcass against the shortening of muscle fibers caused by the sudden drop in temperature on the muscle surface during refrigerated storage, thus reducing the toughness of the meat [69].

Priolo et al. [47] carried out similar work and evaluated lambs raised on natural pastures in France or raised in stalls and fed concentrates to achieve the same rates of growth. They found that meat from stall-fed lambs was more tender and juicier than meat from grass-fed animals. Therefore, it is difficult to make concrete statements about the palatability quality of lamb meat because its acceptability is influenced by the cultural and consumption habits of the test panel [70]. 

The characteristic flavor was also more intense for meat from the LL treatment. This attribute was classified as slightly intense, resulting in tastier meat products and differing from the Control and HL diets, which scored neither intense nor bland. The flavor of red meat largely depends on the animal’s diet. Meat derived from pasture-based production systems incorporates extrinsic characteristics that consumers value while lamb fed on pastures has a more intense flavor than lamb fed on concentrate [71,72].

However, there are also differences depending on the type of pasture [70] because specific compounds in the diet may affect meat quality directly if they are transferred to the meat [73]. The forage diversity of the Pampa Biome offers animals a varied diet that confers variable chemical and sensory characteristics to their meat [74,75,76]. Farias et al. [77] carried out work on consumer perception of beef from cattle raised in native grasslands in the Pampa Biome in which consumers participated in a sensory analysis by word association, and the most cited words were terms related to the categories flavor, texture, aroma, and appearance.

Although meat flavor intensity varies according to production systems [78], normally the most intense flavor is also related to the treatment with a greater amount of fat (IMF, SFT, or carcass fatness). Species-specific meat flavors come from lipids, and they may contain fat-soluble volatile compounds, or be precursors of certain aromatic compounds derived from lipid oxidation [79]. In this study, differences in the subcutaneous fat content of samples from different treatments may be implicated in the expression of greater intensities of lamb flavors. Resconi et al. [79] also found that the intensity of the lamb flavor correlated with the proportions of subcutaneous fat. 

Meat palatability for human consumers is defined as the eating qualities of the meat and is related to consumer acceptance, with tenderness, juiciness, and flavor being the main attributes [69]. In this work, meat from animals finished on LL natural pasture was the most palatable and preferred by consumers. Wang et al. [80] identified more categories of volatile compounds in meat from grazing lambs than from indoor lambs, also demonstrating that grazing time can provide meat with different flavors to consumers.

## 4. Conclusions

We found that finishing lambs on natural pasture is a viable alternative to improve the oxidative stability of the meat, due to an increase in the deposition of muscle α-tocopherol and a decrease in lipid oxidation after 9 d of exposure to oxygen. The Control treatment showed higher cold carcass yield, which is important to producer income, but the meat had a lower WHC and higher cooking loss. Pasture-based diets enhanced OBCFAs, trans vaccenic acid (a precursor to CLA), total CLAs, and decreased elaidic acid.

Lamb carcasses from the LL treatment presented greater STF and better sensorial quality (tenderness, juiciness, and flavor), a positive for consumer sensory perception. The main differences found in FA acid composition were between the Control and HL: HL had higher 8:0, 18:0 (stearic acid), and 6t-18:2 and lower 4:0, 10:0, total MUFAs, 9c-18:1 (oleic acid), and ∆9-desaturase 18 activity than the Control treatment. Despite this, the higher proportion of *D. incanum* (CT-rich legume) in the HL diet did not impact meat nutritional quality. Therefore, finishing lambs on natural pastures can produce high-quality lamb meat, with more antioxidants and beneficial fatty-acid content, in addition to preserving and enhancing the sustainable exploitation of native pastures in the Brazilian Pampa Biome. The influence of legumes, especially *D. incanum*, in this ecosystem, needs to be better studied, especially with regard to monitoring the specific amount of this species consumed by sheep.

## Figures and Tables

**Table 1:** Pasture attributes of paddocks on natural pasture in the Pampa Biome, with a low or high proportion of *Desmodium incanum*.

Legume Proportion	Pasture Characteristics				
	FH (cm)	FM (kg DM/ha)	HAR (kg DM/ha/day)	*D. incanum* (kg DM/ha)	*D. incanum* (% Cover)
Low-legume					
January	22.35	2145.33	22.5	100.88	9.24
February	20.16	2501.87	8.81	16.73	2.29
March	19.12	2533.07	35.67	0	1.59
Average	20.38	2393.42	22.32	39.20	4.37
High-legume					
January	19.18	2344.80	4.2	284.07	20.99
February	19.89	2701.87	3.47	294.28	13.42
March	22.99	2645.87	37.9	157.62	7.62
Average	20.82	2564.18	15.84	245.32	14.01

DM: dry matter; FH: forage height; FM: forage mass; HAR: herbage accumulation rate; and *D. incanum*: *Desmodium incanum.*

**Table 2:** Ingredients, chemical composition, and fatty acid composition of the experimental diets, based on Pampa Biome natural pastures with low and high proportions of *Desmodium incanum* and in a stall-fed system with low bioactive compounds (Control).

Item	Treatments			*Desmodium incanum*
	Control	Low-Legume	High-Legume	
Ingredient (% DM)				
Grass hay	72			
Concentrate	28			
Ground corn	59.43			
Soybean meal	37.9			
Calcareous	2.66			
Chemical composition				
Dry matter (% natural matter)	87.34	87.17	86.68	93.1
Crude protein (% DM)	8.38	7.95	8.47	13.3
Ether extract (% DM)	5.3	2.5	2.65	5.9
Neutral detergent fiber (% DM)	47.36	67.08	61.18	44.21
Acid detergent fiber (% DM)	37.70	37.02	34.79	35.04
Acid detergent lignin (ADL; % DM)	7.75	3.77	3.56	11.8
NIDN (% DM)	0.49	1.19	1.18	1.20
ADIN (% DM)	0.70	0.61	0.71	1.31
C%	42.5	43.8	44.05	47.25
Digestible organic matter	54.07	47.15	48.44	
γ-tocopherol (mg/kg DM)	57.45	5.09	6.15	13.77
α-tocopherol (mg/kg DM)	67.09	151.07	158.51	661.31
Total condensed tannin (%)	0	0	1.11	5.31
Binding CTs (mg/g)	0	0	0	34.59
Fatty acid composition				
16:0	16.95	21.13	21.49	20.72
18:0	2.6	2.58	2.73	7.2
18:1 n-9 (oleic)	17.93	2.82	1.25	5.97
18:2 n-6 c (linoleic)	38.30	14.15	14.33	13.99
20:1 cis 11	9.70	37.04	34.21	12.39

Briefly, the two major FAs present in the Control diet were 18:2 n-6 cis, 18:1 n-9, and 16:0, whereas forages contained mainly 20:1 cis 11, 16:0, and 18:2 n-6 cis. ADL: acid detergent lignin; CTs = condensed tannins; DM = dry matter; NIDN = neutral detergent insoluble nitrogen; and ADIN = acid detergent insoluble nitrogen.

**Table 3:** Quantitative descriptive analysis descriptors sheet for lamb meat.

Descriptor	Definition
Lamb aroma intensity	Characteristic odor of the species
Off-aroma	Undesirable or less desirable odors in lamb meat, such as wool, liver, ammonia, rancidity, etc.
Tenderness	Ease of chewing the sample between the molar teeth
Juiciness	Overall juiciness (initial + sustained) perceived during chewing
Lamb flavor intensity	Aroma and taste associated with the species
Fatty flavor intensity	Aroma and taste associated with fat
Off-flavor	Undesirable or less desirable flavor intensity in lamb meat, such as wool, liver, ammonia, rancidity, etc.

**Table 4:** Dietary and fecal carbon isotope ratio (δ^13^C ‰) of lambs gazing natural pasture from the Pampa Biome with a higher or lower proportion of legumes.

	Diet	Feces	Δδ	% C4 Feces	% C3 Feces	Grass Intake (g/d)	Legume Intake (g/d)
Low-legume	−13.81	−15.63	−1.82	88.41	11.59	596.92	81.4
High-legume	−14.53	−19.19	−4.66	65.80	34.20	549.17	282.9

References δ^13^C (‰) of the plant material: −13.81‰ for the Low-legume group and −29.54‰ for the High-legume group. Δδ = difference between feces and diet.

**Table 5:** Carcass characteristics of pasture-fed lambs with a low or high proportion of legume *Desmodium incanum* and in a stall-fed system with low bioactive compounds (Control).

Variables	Treatments			SEM	*p*-Value
	Control	Low-Legume	High-Legume		
Initial live weight (kg)	25.67	30.31	28.71	2.54	0.4196
Slaughter weight (kg)	34.12	34.27	34.99	0.51	0.3724
Dry matter intake (g/d)	885.11 ^a^	678.30 ^b^	832.06 ^a^	31.9	0.0039
Cold carcass weight (kg)	14.66	14.47	14.24	0.35	0.6688
Cold carcass yield (%)	42.83 ^a^	41.78 ^ab^	40.46 ^b^	0.59	0.0493
Conformation (1–5 scale)	3.06	3.35	3.08	0.19	0.4574
Fatness (1–5 scale)	2.77	2.61	2.39	0.17	0.2724
Ribeye area (cm^2^)	19.46	17.50	18.03	1.15	0.4694
SFT (mm)	2.37 ^b^	3.33 ^a^	1.75 ^b^	0.23	0.0007

Means followed by different letters on the same line denote statistical difference (*p* < 0.05). SFT = subcutaneous fat thickness; SEM = standard error of the mean.

**Table 6:** Effects of the diet on meat quality in *Longissimus thoracis et lumborum* muscle of pasture-fed lambs with a low or high proportion of legume *Desmodium incanum* and in a stall-fed system with low bioactive compounds (Control).

Variables	Treatments			SEM	*p*-Value
	Control	Low-Legume	High-Legume		
pH_24h_	5.41	5.41	5.38	0.01	0.5901
L*	43.71	40.40	42.69	0.70	0.1509
a*	7.43	7.86	7.15	0.27	0.5705
b*	8.67	7.24	8.07	0.27	0.0978
WHC (%)	33.90 ^b^	37.39 ^a^	37.25 ^a^	0.59	0.0229
Cooking loss (%)	37.96 ^a^	31.95 ^b^	35.54 ^ab^	0.94	0.0311
Shear force (N)	45.01	32.46	41.48	0.23	0.0711
Protein (%)	21.03	22.23	20.60	0.32	0.0832
Intramuscular fat (%)	2.47	2.72	2.28	0.207	0.691
Cholesterol (mg/100 g)	87.78	77.81	78.00	3.27	0.4000
γ-tocopherol (mg/kg)	0.051	0.033	0.039	0.003	0.1336
α-tocopherol (mg/kg)	1.40 ^b^	5.34 ^a^	5.73 ^a^	0.52	<0.0001

Means followed by different letters on the same line denote statistical difference (*p* < 0.05). L* = lightness; a* = redness; b* = yellowness; WHC = water holding capacity; and SEM = standard error of the mean.

**Table 7:** Effect of finishing diets on the fatty acid composition (% of total fatty acids) of *longissimus thoracis et lumborum* muscle from lambs based on Pampa Biome natural pastures with low and high proportions of *Desmodium incanum* and in a stall-fed system with low bioactive compounds (Control).

Fatty Acid	Treatments			SEM	*p*-Value
	Control	Low-Legume	High-Legume		
SFAs	44.33	46.49	47.19	0.532	0.076
4:0	0.94 ^a^	0.86 ^ab^	0.39 ^b^	0.095	0.027
8:0	0.02 ^b^	0.03 ^ab^	0.05 ^a^	0.005	0.041
10:0	0.15 ^a^	0.12 ^ab^	0.10 ^b^	0.008	0.043
12:0 (lauric)	0.18	0.28	0.24	0.018	0.065
14:0 (myristic)	2.36	3.11	2.68	0.165	0.202
16:0 (palmitic)	23.22	21.83	21.63	0.323	0.101
18:0 (stearic)	17.79 ^b^	20.28 ^ab^	21.68 ^a^	0.634	0.037
OBCFAs	1.62 ^b^	2.46 ^a^	2.40 ^a^	0.092	<0.0001
15:0	0.31 ^b^	0.56 ^a^	0.53 ^a^	0.027	<0.0001
17:0	0.90 ^b^	1.10 ^a^	1.05 ^a^	0.028	0.008
19:0	0.05 ^b^	0.11 ^a^	0.12 ^a^	0.009	0.008
BCFAs	0.41 ^b^	0.80 ^a^	0.82 ^a^	0.043	<0.0001
*i*-14:0	0.03 ^b^	0.08 ^a^	0.09 ^a^	0.006	<0.0001
*ai*-15:0	0.14 ^b^	0.28 ^a^	0.28 ^a^	0.016	<0.0001
*i*-15:0	0.11 ^b^	0.18 ^a^	0.18 ^a^	0.009	<0.0001
*i*-16:0	0.12 ^b^	0.19 ^a^	0.20 ^a^	0.009	<0.0001
*i*-17:0	0.01 ^b^	0.07 ^a^	0.07 ^a^	0.006	<0.0001
MUFAs	43.47 ^a^	41.07 ^ab^	38.83 ^b^	0.756	0.036
9c-14:1	0.09	0.12	0.11	0.006	0.161
10c-15:1	0.007	0.003	0.007	0.001	0.404
9c-16:1	1.69 ^a^	1.52 ^a^	0.89 ^b^	0.121	0.008
9c-17:1	0.42 ^a^	0.33 ^b^	0.30 ^b^	0.016	0.004
9c-18:1 (oleic)	32.32 ^a^	28.94 ^ab^	26.93 ^b^	0.750	0.008
11c-20:1	0.47 ^b^	1.08 ^a^	1.12 ^a^	0.099	0.009
9t-16:1	0.30 ^b^	0.42 ^a^	0.38 ^a^	0.015	0.002
9t-18:1 (elaidic)	0.13 ^a^	0.08 ^b^	0.08 ^b^	0.008	0.006
10t-18:1	0.09 ^b^	0.21 ^a^	0.20 ^a^	0.015	0.0006
11t-18:1 (vaccenic)	0.90 ^b^	2.38 ^a^	2.53 ^a^	0.162	<0.0001
PUFAs	7.28	5.91	7.88	0.562	0.341
n-6 PUFA	2.68	2.87	3.44	0.180	0.194
6c-18:2 (linoleic)	2.52	2.53	3.29	0.183	0.129
6t-18:2	0.05 ^b^	0.09 ^ab^	0.10 ^a^	0.007	0.012
18:3 n-6	0.02	0.01	0.01	0.004	0.838
20:2 n-6	0.02	0.01	0.02	0.003	0.636
20:3 n-6	0.06	0.15	0.03	0.028	0.218
22:4 n-6	0.01	0.13	0.002	0.051	0.586
n-3 PUFA	2.51	1.81	2.51	0.224	0.351
18:3 n-3 (ALA)	0.11	0.10	0.14	0.017	0.510
20:3 n-3	1.67	1.00	1.54	0.159	0.208
22:5 n-3 (DPA)	0.61	0.58	0.69	0.058	0.708
22:6 n-3 (DHA)	0.14	0.13	0.13	0.015	0.939
CLAs	0.52 ^b^	0.91 ^a^	0.74 ^a^	0.046	0.0009
9c,11t- (rumenic)	0.43 ^c^	0.78 ^a^	0.59 ^b^	0.039	0.0001
10t,12c-	0.007	0.01	0.01	0.002	0.612
11c,9t-	0.06	0.10	0.12	0.012	0.167
11c,13t-	0.03	0.01	0.02	0.003	0.429
Non-conjugated-dienes (18:2)	0.10 ^b^	0.16 ^a^	0.14 ^ab^	0.009	0.009
9c,12t-	0.01	0.03	0.02	0.003	0.426
t9,12c-	0.02 ^b^	0.05 ^a^	0.03 ^ab^	0.004	0.026
8t,13c-	0.009	0.01	0.01	0.002	0.978
10t,15c-/11t,15c-	0.05	0.08	0.07	0.006	0.112
Ratios and indexes					
PUFAs/SFAs	0.16	0.12	0.16	0.013	0.327
n-6/n-3	1.14	2.07	1.43	0.194	0.152
Atherogenicity index (AI)	0.68	0.75	0.73	0.019	0.272
Thrombogenicity index (TI)	1.39	1.54	1.54	0.037	0.178
Desirable fatty acids (DFAs)	68.55	67.26	68.38	0.386	0.354
∆9-desaturase C18	64.53 ^a^	58.75 ^ab^	55.36 ^b^	1.348	0.015

SFAs, saturated fatty acids (10:0; 12:0; 14:0; 16:0; 18:0; 20:0; 22:0, 24:0); OBCFAs, odd- and branched-chain fatty acids (15:0; 17:0; i-14:0; ai-15:0; i-15:0; i-16:0; i-17:0); MUFAs, monounsaturated fatty acids (9c-14:1; 10c-15:1; 7c-16:1; 9c-16:1; 10c-16:1; 11c-16:1; 9c-17:1; 10c-17:1; 11c-17:1; 9c-18:1; 11c-18:1; 12c-18:1; 13c-18:1; 9,15c-19:1; 13c-19:1; 8c-20:1; 11c-20:1; 15c-24:1; 9t-16:1; 9t-18:1; 10t-18:1; 11t-18:1; 12t-18:1). PUFAs, polyunsaturated fatty acids (9t,12c-18:2; 20:3 n-9+ n3+ n6); n-6 PUFA (c-18:2 n-6; t-18:2 n-6; 18:3 n-6; 20:2 n-6; 20:3 n-6; 22:4 n-6); n-3 PUFA (18:3 n-3, 20:3 n-3; 22:5 n-3; 22:6 n-3); CLAs, conjugated linoleic acids (9c,11t; 10t,12c; 11c,9t; 11c,13t); and SEM = standard error of mean. Means followed by different letters on the same line denote statistical difference (*p* < 0.05).

**Table 8:** Average scores (9 cm scale) of the sensory attributes of the *Longissimus thoracis et lumborum* muscle of pasture-fed lambs on natural pasture with a low or high proportion of legume *Desmodium incanum* and in a stall-fed system with low bioactive compounds (Control).

Attribute	Treatments			SEM	*p*-Value
	Control	Low-Legume	High-Legume		
Characteristic aroma ^1^	4.18	4.30	4.29	0.15	0.9347
Off-aroma ^1^	1.04	1.03	1.10	0.17	0.9830
Tenderness ^2^	5.44 ^b^	6.57 ^a^	5.50 ^b^	0.12	<0.0001
Juiciness ^2^	5.47 ^b^	6.49 ^a^	5.54 ^b^	0.12	0.0002
Characteristic flavor ^1^	5.58 ^b^	6.63 ^a^	5.53 ^b^	0.12	<0.0001
Fatty flavor ^1^	1.37	1.52	1.43	0.20	0.9608
Off-flavor ^1^	1.32	1.04	0.89	0.20	0.6774

Means followed by different letters on the same line denote statistical difference (*p* < 0.05). ^1^ Sensory scores (trained panel): (1: none; 9: intense). ^2^ Sensory scores (trained panel): (1: extremely dry, extremely hard; 9: extremely juicy, extremely tender). SEM = standard error of the mean.

## Data Availability

The original contributions presented in this study are included in the article. Further inquiries can be directed to the corresponding author/s.
